# CB_1_ receptor activation in the rat paraventricular nucleus induces bi-directional cardiovascular effects via modification of glutamatergic and GABAergic neurotransmission

**DOI:** 10.1007/s00210-016-1302-y

**Published:** 2016-09-22

**Authors:** Emilia Grzęda, Eberhard Schlicker, Marek Toczek, Iwona Zalewska, Marta Baranowska-Kuczko, Barbara Malinowska

**Affiliations:** 1 Department of Experimental Physiology and Pathophysiology, Medical University of Białystok, Białystok, Poland; 2 Department of Pharmacology and Toxicology, University of Bonn, Bonn, Germany

**Keywords:** Angiotensin AT_1_ receptor, β_2_-adrenoceptor, Cannabinoid CB_1_ receptor, GABA_A_ receptor, NMDA receptor, Paraventricular nucleus of hypothalamus

## Abstract

We have shown previously that the cannabinoid receptor agonist CP55940 microinjected into the paraventricular nucleus of the hypothalamus (PVN) of urethane-anaesthetized rats induces depressor and pressor cardiovascular effects in the absence and presence of the CB_1_ antagonist AM251, respectively. The aim of our study was to examine whether the hypotension and/or hypertension induced by CP55940 given into the PVN results from its influence on glutamatergic and GABAergic neurotransmission. CP55940 was microinjected into the PVN of urethane-anaesthetized rats twice (S_1_ and S_2_, 20 min apart). Antagonists of the following receptors, NMDA (MK801), β_2_-adrenergic (ICI118551), thromboxane A_2_–TP (SQ29548), angiotensin II–AT_1_ (losartan) or GABA_A_ (bicuculline), or the NO synthase inhibitor L-NAME were administered intravenously 5 min before S_2_ alone or together with AM251. The CP55940-induced hypotension was reversed into a pressor response by AM251, bicuculline and L-NAME, but not by the other antagonists. The CP55940-induced pressor effect examined in the presence of AM251 was completely reversed by losartan, reduced by about 50–60 % by MK801, ICI118551 and SQ29548, prevented by bilateral adrenalectomy but not modified by bicuculline and L-NAME. Parallel, but smaller, changes in heart rate accompanied the changes in blood pressure. The bi-directional CB_1_ receptor-mediated cardiovascular effects of cannabinoids microinjected into the PVN of anaesthetized rats depend on stimulatory glutamatergic and inhibitory GABAergic inputs to the sympathetic tone; the glutamatergic input is related to AT_1_, TP and β_2_-adrenergic receptors and catecholamine release from the adrenal medulla whereas the GABAergic input is reinforced by NO.

## Introduction

Cannabinoids act mainly via CB_1_ and CB_2_ receptors and influence multiple functions of the organism (for review, see Pertwee et al. 2010). Their complex cardiovascular effects are related to various peripheral and central mechanisms (for review, see Malinowska et al. 2012; Ibrahim and Abdel-Rahman 2014). Their most pronounced cardiovascular effect in *anaesthetized* rodents is a prolonged hypotension accompanied by a decrease in heart rate (HR) mediated mainly by peripheral presynaptic CB_1_ receptors on sympathetic nerve endings innervating resistance vessels and heart (Malinowska et al. 2010, 2012; Kwolek et al. 2005; Niederhoffer et al. 2003). The peripherally restricted CB_1_ antagonist AM6545 reversed the decreases in blood pressure (BP) and HR elicited by the intravenous injection (i.v.) of the cannabinoid agonist CP55940 into increases (Grzęda et al. 2015). Central CB_1_ receptors are also involved in the cannabinoid-induced decrease in BP in spontaneously hypertensive rats (SHR). Thus, the effect of AM3506 (which inhibits fatty acid amide hydrolase (FAAH), the main hydrolytic enzyme for the endocannabinoid anandamide (AEA)) was reduced by the brain-penetrant CB_1_ receptor antagonists rimonabant or AM251, but not by AM6545 i.v*.* (Godlewski et al. 2010).

The most distinct cardiovascular response to cannabinoids in *conscious* rodents is the pressor response (Gardiner et al. 2009), which can also be detected in anaesthetized rodents as the rapid phase that precedes the prolonged hypotension (e.g. Kwolek et al. 2005; Malinowska et al. 2010, 2012). This stimulatory effect, which has not been fully disclosed so far, is related to a central site of action. Thus, CB_1_ receptor-dependent increases in BP, plasma noradrenaline levels and/or renal sympathetic nerve activity were observed after intracisternal injection of WIN55212-2 and CP55940 to conscious rabbits (Niederhoffer and Szabo, 2000) and rats (Ibrahim and Abdel-Rahman 2011) and after injection of AEA, WIN55212-2 or HU-210 into the cisternal system (Pfitzer et al. 2004), the rostral ventrolateral medulla (RVLM) (Padley et al. 2003) or the dorsal periaqueductal gray (dPAG) (Dean 2011) of anaesthetized rats.

The paraventricular nucleus of the hypothalamus (PVN) represents one of the major integrative sites involved in the control of autonomic cardiovascular responses in the brain (Pyner 2009; Ferguson et al. 2008; Kc and Dick 2010). We found that AEA, its stable analogue methanandamide (MethAEA) or CP55940 injected intracerebroventricularly (i.c.v.) (Malinowska et al. 2010) or into the PVN (Grzęda et al. 2015) decreased BP and/or HR in urethane-anaesthetized rats. However, in the presence of the CB_1_ receptor antagonist AM251 i.v., the cardiodepressor effects of cannabinoids (given i.c.v. or into the PVN) were reversed into pure pressor and tachycardic responses, which were inhibited by the local microinjection of AM251. The CB_2_ receptor antagonist SR144528 i.v. did not modify the cardiovascular effects of CP55940 given into the PVN. Bilateral PVN lesion with kainic acid abolished pressor and depressor responses to CP55940 (Grzęda et al. 2015).

Cannabinoid CB_1_ receptors are located mainly presynaptically inhibiting the release of various neurotransmitters including glutamate and GABA (Schlicker and Kathmann 2001). Regulation of food intake and energy homeostasis (Busquets-Garcia et al. 2015) and stress response (Senst and Bains 2014) are influenced in opposite direction by CB_1_ receptors in the PVN according to their localization on different types of neurons (e.g. glutamatergic and GABAergic). Similarly, the sympathetic tone controlling the cardiovascular system results from the direct balance between stimulatory and inhibitory inputs, depending on glutamatergic and GABAergic neurotransmission in the PVN (Fig. 5), respectively. Thus, it is possible that the bi-directional CB_1_ receptor-mediated cardiovascular effects of CP55940 given into the PVN (Grzęda et al. 2015) result from the CB_1_ receptor-dependent modification of glutamatergic and GABAergic neurotransmission in the PVN. We have previously shown that the increase in BP induced by AEA (i.c.v.) was mediated by central β_2_-adrenergic, *N*-methyl-d-aspartate (NMDA) and thromboxane A_2_ (TXA_2_) TP receptors (Malinowska et al. 2010). The two main neurotransmission systems in the PVN are indirectly modified e.g. by β_2_, TP and angiotensin II (Ang II) AT_1_ receptors and by nitric oxide (NO) (Pyner 2009; Ferguson et al. 2008; Kc and Dick 2010). Thus, the aim of our study performed on urethane-anaesthetized rats was to examine whether the depressor and/or pressor cardiovascular effects induced by CB_1_ receptor activation by CP55940 in the PVN results from its influence on glutamatergic and GABAergic neurotransmission and/or additional factors (β_2_, TP and AT_1_ receptors as well as NO) indirectly modifying the latter two systems.

## Materials and methods

Male normotensive Wistar rats (weighing 280–350 g) with free access to food pellets and water were used. All surgical procedures and experimental protocols were in accordance with European and Polish legislation and were approved by the local Animal Ethics Committee in Białystok (Poland).

### Placement of a cannula for drug administration into the PVN

Rats were anaesthetized intraperitoneally (i.p.) with pentobarbitone sodium (300 μmol/kg) and placed in a stereotaxic instrument (Stoelting WPI, Wood Dale, IL, USA). Stainless cannulae (outer and inner diameter of 0.5 and 0.3 mm, respectively) were stereotaxically implanted on the right side. The coordinates for the PVN were 1.5 mm caudal to the bregma, 0.5 mm lateral to the midline and 8 mm below the skull surface. Cannulae were fastened to the skull with acrylic cement. Rats were protected against infections by topical administration of the antibiotic doxycycline. The rats were then returned to their individual cages and allowed to recover.

### Anaesthetized rats

At least 7 days later, rats were anaesthetized i.p. with urethane (14 mmol/kg). The trachea was cannulated. Systolic BP (SBP), mean BP (MBP) and diastolic BP (DBP) were measured from the right carotid artery via a transducer (ISOTEC; Hugo Sachs Elektronik–Harvard Apparatus GmbH, March, Germany). HR was recorded from the ECG by means of subcutaneous electrodes. Body temperature was maintained constant at approximately 37 °C using a heating pad (Bio-Sys-Tech, Białystok, Poland) and monitored by a rectal probe transducer (Physitemp BAT10; Physitemp Instruments, Inc., Clifton, NJ, USA). The left femoral vein was cannulated for i.v. injection of drugs administered in a volume of 0.5 mL/kg. The right femoral vein was prepared for infusion of prostaglandin F_2α_ (PGF_2α_) by means of a Graseby 3100 syringe pump (Graseby Medical, Watford, Herts, UK). After surgical procedures, animals were gently placed on their abdomen and cardiovascular parameters were allowed to stabilize. Twenty minutes later, experiments were performed.

### Experimental protocol

Agonists of NMDA receptors (NMDA; 1 mmol/rat, Kawabe et al. 2008) or cannabinoid receptors (CP55940; 0.1 nmol/rat, Grzęda et al. 2015) were administered into the PVN twice (S_1_ and S_2_, 20 min apart). PVN microinjections were administered slowly in a volume of 100 nL per rat and were completed within 1 min. We recorded agonist-induced maximal decreases or increases in the particular cardiovascular parameters that persisted for at least 5 s. Moreover, the non-selective nitric oxide synthase inhibitor L-NAME 37 μmol/kg (Gordish and Beierwaltes 2014) and the following antagonists were used: MK801 1 μmol/kg (NMDA receptor; Malinowska et al. 2010), ICI118551 1 μmol/kg (β_2_-adrenoceptor; Malinowska et al. 2010), SQ29548 1 μmol/kg (thromboxane A_2_ receptor (TP); Malinowska et al. 2010), losartan 10 μmol/kg (angiotensin II receptor (AT_1_); Kwolek et al. 2005) and bicuculline 5 μmol/kg (GABA_A_ receptor; Elsersy et al. 2006). The latter antagonists/blockers or their solvents were administered i.v. alone or together with the CB_1_ receptor antagonist AM251 3 μmol/kg (Grzęda et al. 2015) 5 min before S_2_. In some experiments, bilateral acute adrenalectomy or a sham operation was performed 10 min before the second CP55940 administration. Adrenalectomy was done through two dorsolateral skin and muscular incisions. The adrenal glands were pulled out by holding the periadrenal fat and then excised. Sham operated animals were handled in the same way except that the adrenals were not removed. L-NAME increased DBP to about 80–90 mmHg. Since this effect did not recover during S_2_ and since the amplitude of vasopressor/vasodepressor effects is dependent on the basal BP (e.g. Kwolek et al. 2005), PGF_2α_ (0.17–1.47 μmol/kg/h) was infused to control animals to ensure a basal DBP comparable to that of L-NAME-treated rats. At the end of the experiments, correct cannula placement was confirmed histologically and analysed by light microscopy. Only animals for which the correct placement of the guide cannula to the PVN was confirmed were included in this study.

## Data analysis

Results are given as means ± SEM; *n* refers to the number of rats. In order to quantify the effects of antagonists on the cardiovascular effects of NMDA or CP55940, the agonist-induced maximal decreases or increases in BP and HR during S_1_ and S_2_ were calculated as percentage of the respective basal SBP, DBP, MBP and HR immediately before injection of the particular agonist. This procedure was chosen to minimize the influence of natural inter-subject variability on final data. For comparison of the mean values, the *t* test for paired and unpaired data was used, as appropriate. When two or more groups were compared with the same control, one-way analysis of variance (ANOVA) followed by Dunnett test was used. Differences were considered as significant when *P* < 0.05.

### Drugs

AM251 [(*N*-(piperidin-1-yl)-5-(4-iodophenyl)-1-(2,4-dichlorophenyl)-4-methyl-1H-pyrazole-3-carboxamide)] (Sigma-Aldrich, St. Louis, MO, USA); bicuculline, CP55940 [(−)-*cis*-3-[2-hydroxy-4-(1,1-dimethylheptyl)phenyl]-*trans*-4-(3-hydroxypropyl)cyclohexanol], ICI118551 [(erythro-(±)-1-(7-methylindan-4-yloxy)-3-isopropylaminobutan-2-ol] (Tocris Cookson, Bristol, UK); L-NAME (N^ω^-nitro-l-arginine-methyl ester); losartan monopotassium salt (Cayman Chemicals, Ann Arbor, MI, USA); MK801 [((5*R*,10*S*)-(+)-5-methyl-10,11-dihydro-5H-dibenzo(a,d)cyclohepten-5,10-imine hydrogen maleate], NMDA (*N*-methyl-d-aspartic acid) (Sigma-Aldrich); SQ29548 [([1*S*-[1α, 2α(Z), 3α, 4α]]-7-[3-[[2-[(phenylamino)carbonyl]hydrazino]methyl]-7-oxabicyclo[2.2.1]hept-2-yl]-5-heptenoic acid] (Cayman Chemicals); pentobarbitone sodium (Biowet, Puławy, Poland); PGF_2α_; urethane (Sigma-Aldrich).

Drugs were dissolved in saline with the following exceptions: AM251 in a mixture of ethanol, Cremophor El, DMSO and saline (1:1:1:9.5); SQ29548 in a mixture of saline and DMSO (20:1); CP55940 was dissolved in 19 % solution of cyclodextrin. Solvents for agonists microinjected into the PVN or for particular antagonists/blockers given i.v. did not modify basal BP and HR.

## Results

### General

Basal SBP, DBP, MBP and HR measured immediately before the first (S_1_) and the second (S_2_) microinjection of NMDA or CP55940 into the PVN are given in Table 1. In animals not treated with any receptor antagonists, values of basal cardiovascular parameters were comparable before S_1_ and S_2_, confirming that the cardiovascular effects induced by agonists during S_1_ ceased before S_2_. Basal BP and HR were not altered by i.v. administration of the following antagonists: AM251 (CB_1_ receptors), MK801 (NMDA receptors), losartan (AT_1_ receptors), SQ29548 (TP receptors), ICI118551 (β_2_-adrenoceptors), and bicuculline (GABA_A_ receptors) and by bilateral adrenalectomy. The NO synthesis inhibitor L-NAME given i.v. alone or together with AM251 increased SBP, DBP and MBP by about 20–30 mmHg but did not affect basal HR. In the related control group, PGF_2α_ was infused to adjust basal BP values to those in rats treated with L-NAME (Table 1).

**Table 1:** Basal systolic, diastolic and mean blood pressure (SBP, DBP and MBP in mmHg) and heart rate (HR in beats/min) immediately before S_1_ or S_2_ in urethane-anaesthetized rats

Agonist	AM251	Antagonist (i.v.)	Dose^a^	Number	Before S_1_				Before S_2_			
					SBP	DBP	MBP	HR	SBP	DBP	MBP	HR
NMDA	**−**	−	−	4	121 ± 8	49 ± 4	76 ± 9	376 ± 18	116 ± 14	48 ± 4	74 ± 8	380 ± 18
	**+**	−	−	4	118 ± 5	67 ± 7	79 ± 7	378 ± 14	126 ± 4	60 ± 9	80 ± 7	396 ± 18
CP55940	**+**	Solvent^b^	−	8	87 ± 8	54 ± 4	66 ± 5	334 ± 8	91 ± 7	57 ± 3	65 ± 4	334 ± 7
	−	MK801	1	4	103 ± 5	45 ± 3	60 ± 1	349 ± 18	102 ± 7	47 ± 3	64 ± 3	348 ± 13
	**+**	MK801	1	4	117 ± 7	66 ± 7	85 ± 6	352 ± 9	118 ± 5	66 ± 5	85 ± 3	345 ± 9
	−	ICI118551	1	4	96 ± 6	61 ± 8	75 ± 5	360 ± 6	96 ± 5	60 ± 6	76 ± 5	357 ± 3
	**+**	ICI118551	1	5	87 ± 3	55 ± 2	66 ± 3	353 ± 6	86 ± 3	55 ± 2	66 ± 3	343 ± 9
	−	Losartan	10	4	104 ± 4	65 ± 6	73 ± 7	344 ± 6	101 ± 3	63 ± 5	73 ± 7	343 ± 5
	**+**	Losartan	10	4	101 ± 5	57 ± 4	72 ± 4	329 ± 12	98 ± 5	57 ± 4	72 ± 4	327 ± 12
	−	Bicuculline	5	4	84 ± 2	54 ± 4	66 ± 3	354 ± 8	83 ± 4	52 ± 4	66 ± 3	357 ± 8
	**+**	Bicuculline	5	4	73 ± 2	46 ± 1	59 ± 1	340 ± 26	77 ± 2	48 ± 1	62 ± 2	318 ± 16
	**+**	Solvent^c^	−	4	89 ± 8	56 ± 6	70 ± 6	345 ± 16	93 ± 5	53 ± 4	67 ± 5	343 ± 13
	−	SQ29548	1	4	98 ± 7	57 ± 5	71 ± 5	365 ± 11	96 ± 6	58 ± 4	73 ± 6	367 ± 11
	**+**	SQ29548	1	4	87 ± 6	60 ± 8	72 ± 8	348 ± 17	87 ± 5	60 ± 7	71 ± 7	336 ± 9
	−	L-NAME^d^	27	4	*108 ± 6*	*71 ± 6*	*90 ± 6*	394 ± 25	127 ± 8^+,*^	94 ± 10^+,*^	109 ± 11^+,*^	398 ± 18
	**+**	L-NAME^d^	27	4	*87 ± 3*	*53 ± 4*	*61 ± 1*	334 ± 14	110 ± 6^+^	79 ± 8^+,*^	97 ± 8^+,*^	332 ± 10
	**+**	PGF_2α_^d^	−	4	*90 ± 4*	*58 ± 8*	*64 ± 13*	336 ± 11	115 ± 9^+^	88 ± 9^+,*^	95 ± 7^+,*^	316 ± 4
	**+**	Sham	−	4	78 ± 4	52 ± 2	61 ± 3	335 ± 7	78 ± 3	54 ± 1	64 ± 3	332 ± 6
	**+**	Adrenalectomy	−	4	81 ± 3	45 ± 2	56 ± 3	317 ± 8	80 ± 7	45 ± 3	57 ± 4	324 ± 9

NMDA (1 mmol/rat) or CP55940 (0.1 nmol/rat) was injected into the paraventricular nucleus twice (S_1_-S_2_) 20 min apart. Antagonists or their solvents (alone or in combination with AM251 3 μmol/kg) were given 5 min before S_2_. Data are given as the means ± SEM of *n* experiments

^a^Doses of antagonists/blockers are given in μmol/kg

^b^Solvents for MK801, ICI118551, losartan and bicuculline

^c^Solvent for SQ29548. PGF_2α_ was infused at a dose of 0.17–1.47 μmol/kg/h

^d^Basal SBP, MBP and DBP values before S_2_ (i) are higher (^+^ *P* < at least 0.05) than the respective values before S_1_ and (ii) are higher (*P < at least 0.05) or tend to be higher than the respective values before S_2_ in the presence of solvent^b^ (for the sake of simplicity, comparators are marked with *italics* in the table)

### Influence of the CB_1_ receptor antagonist AM251 on the cardiovascular effects of NMDA and CP55940 given into the PVN

**Fig. 1:**
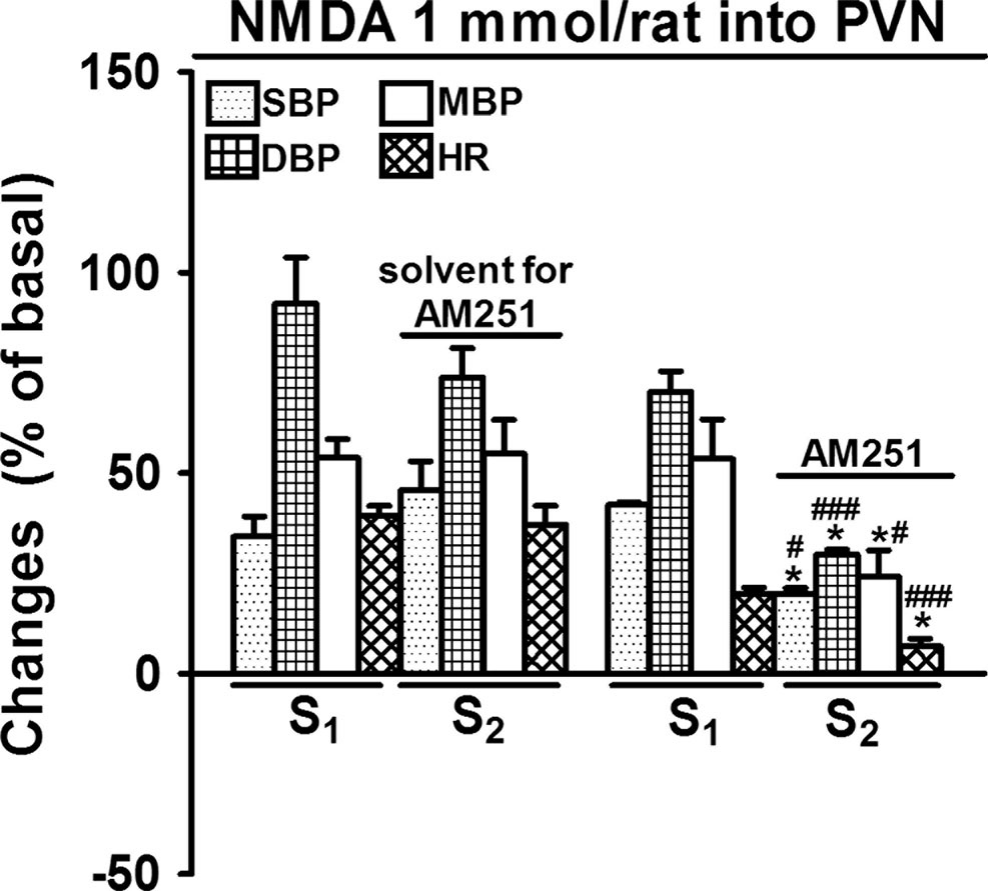
Influence of AM251 on the increases in systolic, diastolic and mean blood pressure (SBP, DBP, MBP) and heart rate (HR) induced by NMDA microinjected into the paraventricular nucleus (PVN) in urethane-anaesthetized rats. NMDA was administered twice (S_1_ and S_2_, 20 min apart). AM251 3 μmol/kg or its solvent was administered i.v. 5 min before S_2_. Results are calculated as percentage of basal values determined immediately before S_1_ and S_2_ (see Table 1). Means ± SEM of 4 rats. *P < 0.001 compared to the corresponding S_1_; ^#^ *P* < 0.05; ^###^ *P* < 0.001 compared to the corresponding S_2_ values without AM251

As shown in Fig. 1, the first (S_1_) microinjection of NMDA (1 mmol/rat) into the PVN increased SBP, DBP, MBP and HR by about 34, 92, 54 and 39 % of basal values (i.e. by 41 ± 6; 45 ± 5 and 41 ± 4 mmHg and 148 ± 10 beats/min; *n* = 4, respectively). The second administration of the agonist (S_2_) into the PVN of the same rat induced comparable increases in BP and HR. The pressor response of NMDA lasted for 550 ± 87 s, and the increase in HR for 724 ± 46 s (*n* = 4). The cannabinoid CB_1_ antagonist AM251 3 μmol/kg i.v. diminished the NMDA-induced increases in SBP, DBP, MBP and HR by about 50–60 % (Fig. 1).

**Fig. 2:**
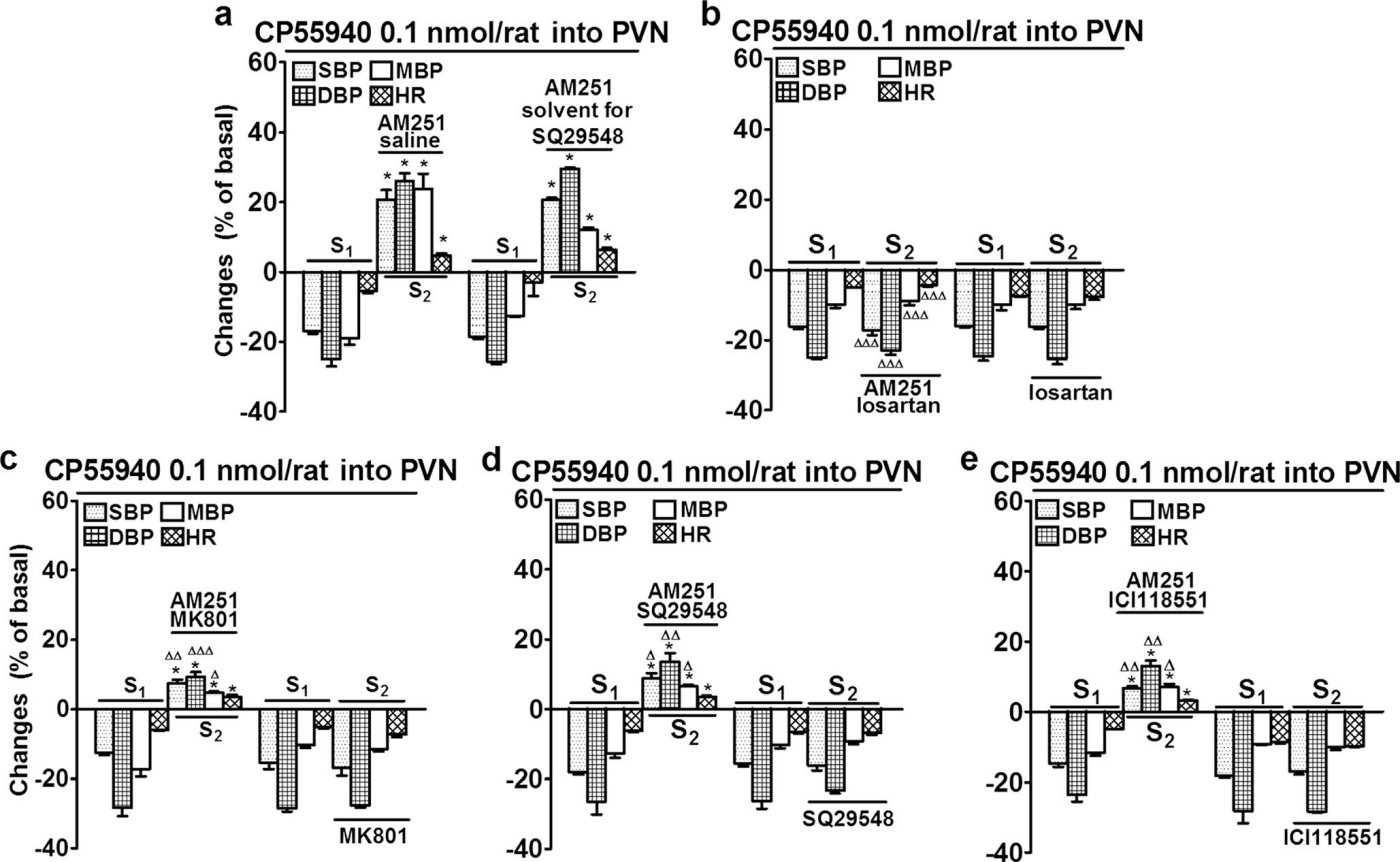
Influence of AM251 alone (**a**) and of AM251 plus losartan (**b**), MK801 (**c**), SQ29548 (**d**) and ICI118551 (**e**) on the increases in systolic, diastolic and mean blood pressure (SBP, DBP, MBP) and heart rate (HR) induced by CP55940 microinjected into the paraventricular nucleus (PVN) in urethane-anaesthetized rats. CP55940 was administered twice (S_1_ and S_2_, 20 min apart). AM251 3 μmol/kg was administered i.v. 5 min before S_2_ either together with saline (solvent for losartan, MK801 and ICI118551) or the solvent for SQ29548 (**a**) or together with losartan 10 μmol/kg, MK801 1 μmol/kg, SQ29548 1 μmol/kg or ICI118551 1 μmol/kg (**b–e**). Results are calculated as percentage of basal values determined immediately before S_1_ and S_2_ (see Table 1). Means ± SEM of 4–8 rats. *P < 0.001 compared to the corresponding S_1_; ^Δ^ *P* < 0.05; ^ΔΔ^ *P* < 0.01; ^ΔΔΔ^ *P* < 0.001 compared to the S_2_ with AM251 and the respective solvent

**Fig. 3:**
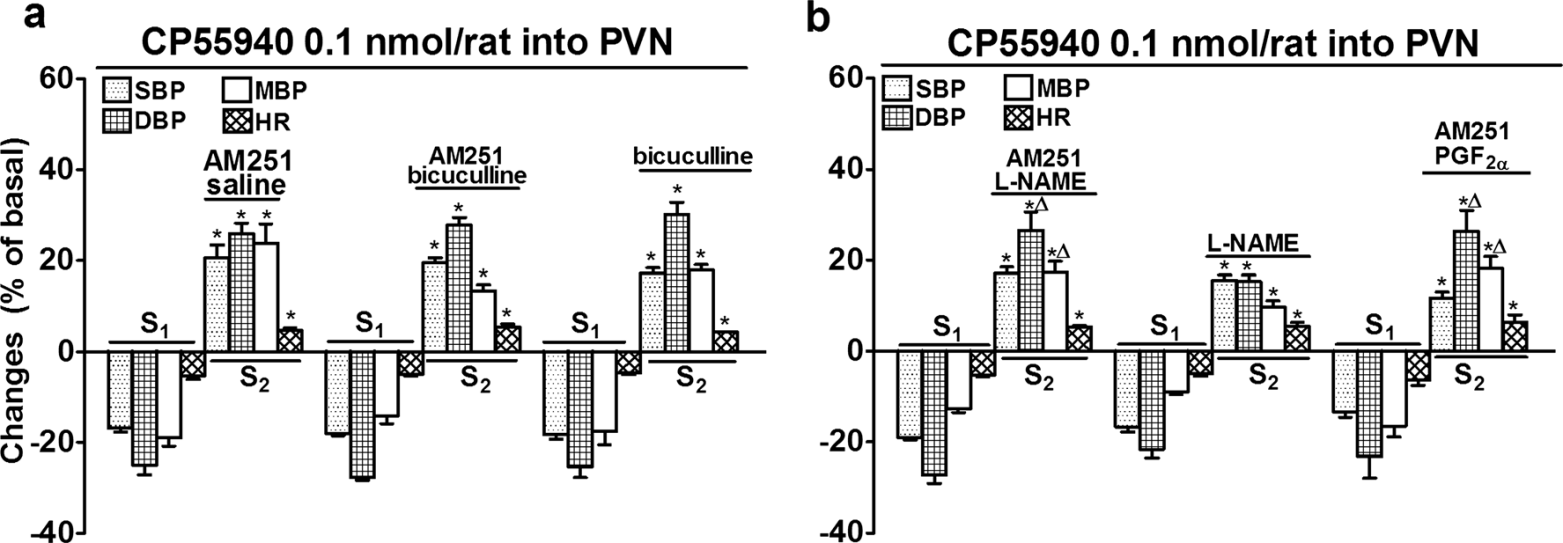
Influence of AM251, bicuculline (**a**) and L-NAME (**b**) on the increases in systolic, diastolic and mean blood pressure (SBP, DBP, MBP) and heart rate (HR) induced by CP55940 microinjected into the paraventricular nucleus (PVN) in urethane-anaesthetized rats. CP55940 was administered twice (S_1_ and S_2_, 20 min apart). AM251 3 μmol/kg [given with saline (solvent for bicuculline, L-NAME and PGF_2α_)], bicuculline 5 μmol/kg and/or L-NAME 37 μmol/kg were administered i.v. 5 min before S_2_. In one series (**b**) with AM251 (but without L-NAME), PGF_2α_ 0.17–1.47 μmol/kg/h was infused to adjust basal parameters before S_2_ to those in rats treated with L-NAME. Results are calculated as percent of basal values determined immediately before S_1_ and S_2_ (see Table 1). Means ± SEM of 4–8 rats. *P < 0.001 compared to the corresponding S_1_ values; ^Δ^ *P* < 0.05 compared to S_2_ with L-NAME only

**Fig. 4:**
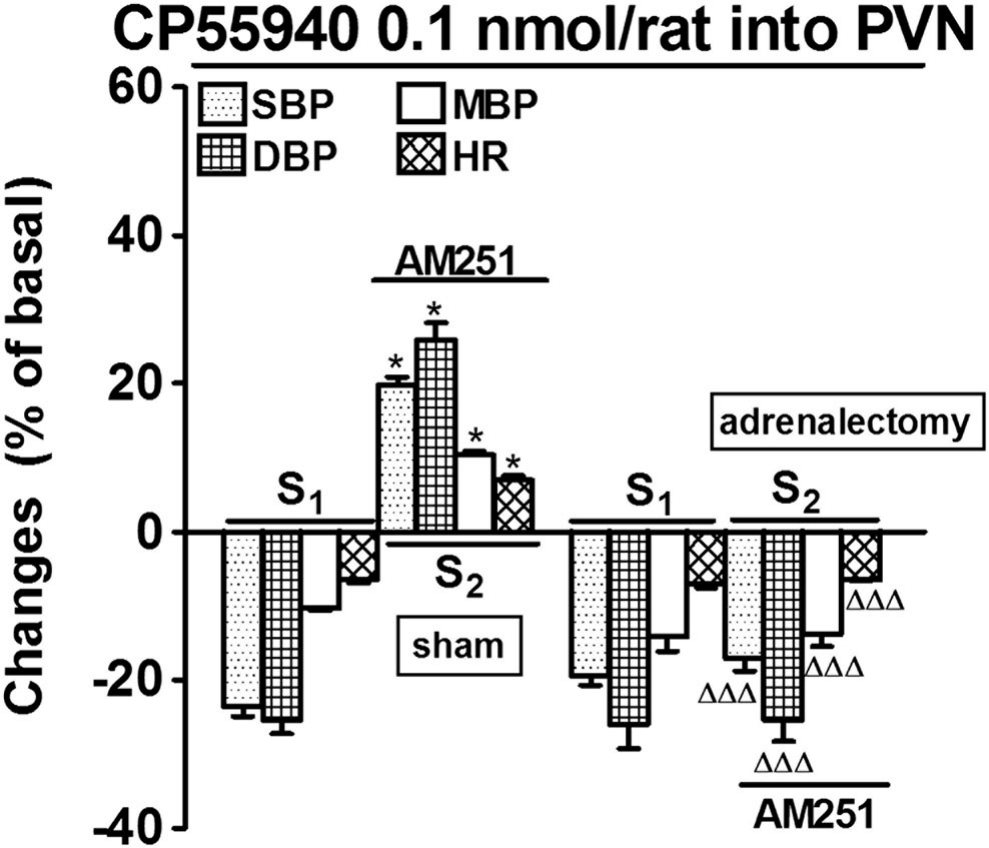
Influence of adrenalectomy on the increases in systolic, diastolic and mean blood pressure (SBP, DBP, MBP) and heart rate (HR) induced by CP55940 microinjected into the paraventricular nucleus (PVN) in urethane-anaesthetized rats. Adrenalectomy or sham operation was performed about 15 min before the second CP55940 microinjection. AM251 3 μmol/kg was given i.v. 5 min before S_2_. Results are calculated as percentage of basal values determined immediately before S_1_ and S_2_ (see Table 1). Means ± SEM of 4 rats. *P < 0.001 compared to the corresponding S_1_; ^ΔΔΔ^ *P* < 0.001 compared to the S_2_ values in sham operated rats

Similarly to our previous observations (Grzęda et al. 2015), the microinjection of CP55940 (0.1 nmol/rat) into the PVN decreased SBP, DBP, MBP and HR during S_1_ by about 15, 25, 20 and 5 % of basal values (i.e. by 16 ± 1; 13 ± 1 and 14 ± 1 mmHg and 17 ± 1 beats/min, respectively; *n* = 8; Fig. 2a). The cardiovascular effects of CP55940 lasted for about 2 min (for details, see Grzęda et al. 2015). Similar decreases in BP and HR were obtained during S_1_ in response to microinjection of CP55940 into the PVN before administration of particular antagonists (see S_1_ in Figs. 2, 3, and 4). However, in the presence of AM251 3 μmol/kg (independent of two various solvents for particular antagonists) i.v*.*, CP55940 increased SBP, DBP, MBP and HR by about 20, 25, 25 and 5 %, (i.e. by 18 ± 2; 14 ± 1 and 15 ± 1 mmHg and 16 ± 1 beats/min, respectively; *n* = 8) (Figs. 2a, 3a, and 4).

### Influence of NMDA, angiotensin AT_1_, thromboxane TP and β_2_-adrenoceptor antagonists on the cardiovascular effects of CP55940

In contrast to AM251, the hypotension and the bradycardia induced by microinjection of CP55940 (0.1 nmol/rat) into the PVN (S_1_) were not modified (see S_2_) by the i.v. administration of antagonists of AT_1_, NMDA, TP receptors and β_2_-adrenoceptors, i.e., losartan (10 μmol/kg; Fig. 2b), MK801 (1 μmol/kg; Fig. 2c), SQ29584 (1 μmol/kg; Fig. 2d) and ICI118551 (1 μmol/kg; Fig. 2e), respectively.

Next, we examined how the pressor and tachycardic effects of CP55940 (0.1 nmol/rat) given into the PVN obtained in the presence of AM251 are affected by the four antagonists. Losartan completely reversed the CP55940-stimulated increases in BP and HR to hypotensive and bradycardic effects which were very similar to the responses induced by CP55940 in the absence of AM251 (Fig. 2b). MK801 (Fig. 2c), SQ29584 (Fig. 2d) and ICI118551 (Fig. 2e) diminished the CP55940-induced increases in SBP, DBP and MBP by about 60–70, 50–60 and 70–80 %, respectively but they did not affect the CP55940-stimulated increases in HR.

### Influence of a GABA_A_ receptor antagonist and an NO synthase inhibitor on the cardiovascular effects of CP55940

The GABA_A_ receptor antagonist bicuculline (5 μmol/kg, i.v.; Fig. 3a) and the NO synthase inhibitor L-NAME (37 μmol/kg, *i.v*.; Fig. 3b) like AM251 reversed the depressant effects of CP55940 (0.1 nmol/rat given into the PVN) on SBP, DBP, MBP and HR into stimulatory ones. The CP55940-induced increases in SBP, DBP and MBP and HR in the presence of bicuculline were not affected when, in addition, AM251 (3 μmol/kg) was given i.v. (Fig. 3a) and they were comparable to those obtained in the presence of AM251 only.

By contrast, the CP55940-induced increases in DBP and MBP (but not those in SBP and HR) obtained in the presence of L-NAME were by about 45 and 65 % lower than those determined in the presence of AM251 (compare Fig. 3a, b). Moreover, the CB_1_ receptor antagonist enhanced the CP55940-induced increases in DBP and MBP (but not those in SBP and HR) obtained in the presence of L-NAME by about 75 and 80 %, respectively (Fig. 3b). Since L-NAME increased the level of the basal BP by about 20–30 mmHg (Table 1), additional experiments were carried out in which the baseline level of DBP was increased to about 90 mmHg by i.v. infusion of PGF_2α_. Similar effects of AM251 on the CP55940-induced increases in BP and HR occurred in the absence (Fig. 3a) and presence of PGF_2α_ (Fig. 3b). PGF_2α_ plus AM251 enhanced the CP55940-induced increases in DBP and MBP (but not those in SBP and HR) obtained in the presence of L-NAME by about 70 and 85 %, respectively (Fig. 3b).

### Influence of bilateral adrenalectomy on the cardiovascular effects of CP55940

As shown in Fig. 4, bilateral adrenalectomy did not affect the hypotensive and bradycardic responses to CP55940 (0.1 nmol/rat) given into the PVN but completely prevented the pressor and tachycardic effects of CP55940 (given into the PVN) observed after previous i.v. administration of AM251. In sham operated animals, the CP55940-induced increases in SBP, DBP and MBP and HR in the presence of AM251 were similar to the changes observed under control conditions (compare Figs. 4, 2a, and 3a).

## Discussion

### General

The present study was carried out to clarify whether the hypotension and/or hypertension induced by CP55940 given into the PVN results from its influence on the two main neurotransmission systems (glutamatergic and GABAergic) and/or is related to additional receptors and NO indirectly modifying the two above systems. We performed experiments on anaesthetized rats to continue our previous experiments (Malinowska et al. 2010; Grzęda et al. 2015) and to reduce stress since the activation of CB_1_ receptors inhibits the stress-relevant neurons in the PVN (Crosby and Bains 2012). Like in our previous studies, urethane was used as anaesthetic and CP55940 served as cannabinoid receptor agonist.

Antagonists were administered i.v. since only a small volume could be microinjected into the PVN and only in this way, we were able to examine the CP55940-induced decreases (S_1_) and increases (S_2_) in cardiovascular parameters in one rat. On the other hand, this route of administration does not allow us to exclude that the effects of the antagonists may occur on central sites apart from the PVN. The effectiveness of the antagonists on centrally mediated effects after their i.v. application was confirmed in our previous publications (MK801, ICI118551 and SQ29548; Malinowska et al. 2010; Grzęda et al. 2015) or by other investigators (L-NAME, bicuculline and losartan; Turnbull et al. 1998; Elsersy et al. 2006; Busnardo et al. 2014). Moreover, (1) we have shown previously that central but not peripheral NMDA, β_2_ and TP receptors are involved in the pressor effect of cannabinoids since the effects of AEA or MetAEA given i.v. were reduced by NMDA, β_2_ and TP receptor antagonists in “intact” but not in pithed rats (i.e. in a model in which the effects of drugs involve peripheral sites only; Kwolek et al. 2005; Malinowska et al. 2010). (2) The pressor responses to AEA i.c.v. (obtained in the presence of a CB_1_ antagonist) were diminished by i.v. administration of NMDA, β_2_ and TP antagonists (Malinowska et al. 2010). (3) The AT_1_ receptor antagonist losartan i.v. did not modify the cardiovascular effects of AEA i.v. (including its pressor effect; Kwolek et al. 2005). Moreover, subcutaneous injection of losartan inhibits AT_1_ receptors (labelled by ^125^I-Ang II binding) in the rat PVN (Wang et al. 2003). To the best of our knowledge, peripheral GABA_A_ receptors do not participate in the regulation of the rat cardiovascular system.

### Modification of the CP55940-induced hypotension and bradycardia

**Fig. 5:**
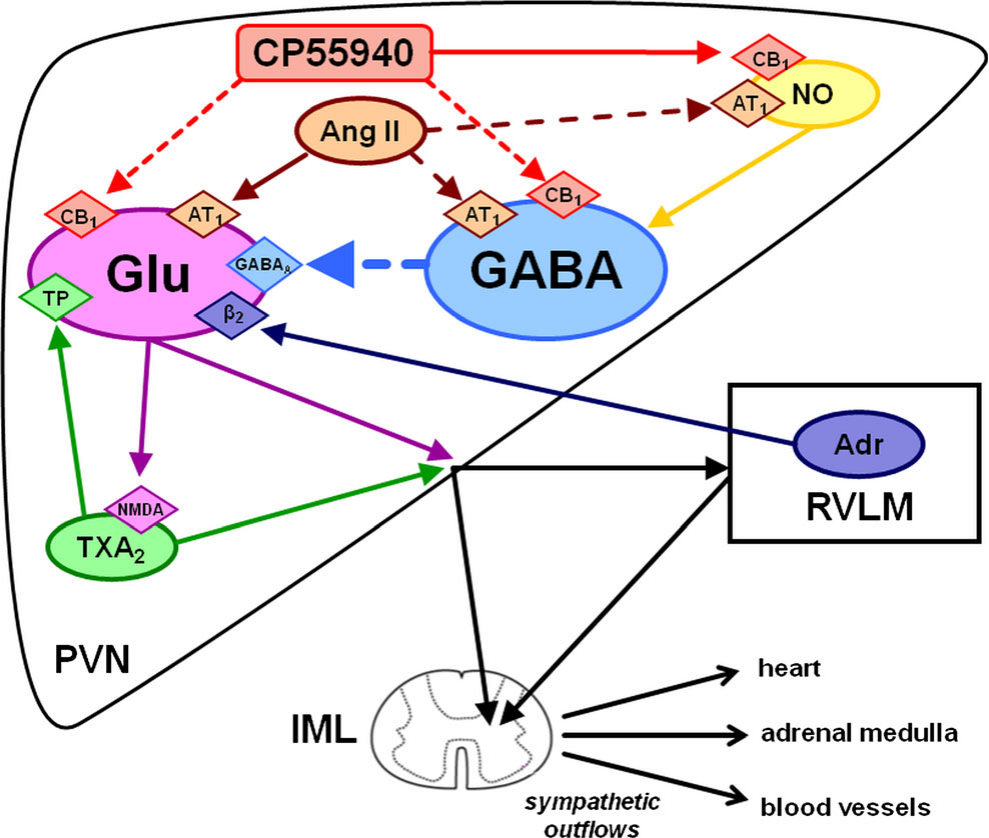
Possible mechanisms involved in the effect of the CB receptor agonist CP55940 topically administered to the paraventricular nucleus (PVN) on the sympathetic outflow and cardiovascular parameters. Activation of presynaptic inhibitory CB_1_ receptors on glutamatergic (Glu) neurones leads to a decrease in the sympathetic outflow (mainly to resistance vessels and heart) and a fall in blood pressure. Activation of presynaptic inhibitory CB_1_ receptors on GABAergic (γ-aminobutyric acid) neurons leads to an increase in the sympathetic outflow (mainly of the adrenal medulla) and in blood pressure. Activation of presynaptic facilitatory (i) angiotensin II (Ang II) AT_1_, (ii) thromboxane A_2_ (TXA_2_) TP and (iii) adrenaline (Adr) β_2_-adrenergic receptors increases whereas activation of GABA_A_ receptors decreases the release of glutamate. Nitric oxide (NO) stimulates GABA release. For the sake of clarity, only interactions examined in the present study have been shown in this scheme. An inhibitory effect of Ang II and a facilitatory effect of CP55940 on NO production although opposite in direction to the effects usually obtained by AT_1_ and CB_1_ receptor activation, respectively, are also indicated. stimulatory inputs;  inhibitory inputs. *RVLM* rostral ventrolateral medulla, *IML* intermediolateral column

Two main observations are in line with our working hypothesis that the decrease in BP and HR in response to the microinjection of CP55940 into the PVN is related to the activation of presynaptic inhibitory CB_1_ receptors on glutamatergic neurons. Firstly, we confirmed that AM251 i.v. reversed the depressant effects of CP55940 on BP and HR into stimulatory ones (Grzęda et al. 2015). We cannot exclude the possibility that the effect of AM251 i.v. is partially related to the inhibition of peripheral presynaptic inhibitory CB_1_ on sympathetic nerve endings. However, the pressor effect of CP55940 given into the PVN in the presence of AM251 i.v. was completely reversed by AM251 microinjected into the PVN (Grzęda et al. 2015). The final integration of the sympathetic outflow by the PVN results from the balance between stimulatory and inhibitory inputs depending on glutamate and GABA, respectively (Fig. 5; Pyner 2009; Ferguson et al. 2008; Kc and Dick 2010). In the PVN, presynaptic inhibitory CB_1_ receptors are localized both on glutamatergic and GABAergic synapses (Senst and Bains 2014); activation of CB_1_ receptors on GABAergic neurons should result in hypertension. The question arises why in our hands CP55940 primarily activates CB_1_ receptors on glutamatergic neurons? Different feeding (Busquets-Garcia et al. 2015) or emotional states (Senst and Bains 2014) determine the direction of final effects of endocannabinoids on CB_1_ receptors on glutamatergic and GABAergic transmission in the PVN on food intake or stress. In our previous paper (Grzęda et al. 2015), we suggested that the higher resting sympathetic tone in urethane-anaesthetized rats (Carruba et al. 1987) may lead to a stronger inhibitory effect of cannabinoids on glutamate than on GABA release.

Secondly, similarly to AM251 (present paper and Grzęda et al. 2015), blockade of GABA_A_ receptors and NO synthesis, i.e. of mechanisms that have an inhibitory influence on the glutamatergic neurotransmission (Pyner 2009; Kc and Dick 2010), reversed the CP55940-induced hypotension and bradycardia into pressor and tachycardic responses. As shown in Fig. 5, GABA inhibits the stimulatory glutamatergic influence via GABA_A_ receptors (Li et al. 2006) and NO potentiates the inhibitory effect of GABAergic transmission (Pyner 2009; Kc and Dick 2010). Thus, the respective blockers bicuculline and L-NAME dis-inhibited the effects induced by GABA and NO and reversed the depressor influence of CP55940 on cardiovascular parameters into a stimulatory one. The type of NO synthase is unclear since L-NAME is a non-selective inhibitor and both neuronal and endothelial NO synthase in the PVN are involved in the modulation of the sympathetic tone (Lu et al. 2015).

Bilateral adrenalectomy did not affect the CP55940-induced hypotension and bradycardia. These results are in line with our previous study and allow us to exclude the possibility that catecholamines released from the adrenal medulla contributed to the neurogenic cardiovascular responses (Malinowska et al. 2001).

### Modification of the CP55940-induced pressor and tachycardic responses

The pressor responses to CP55940 given into the PVN were routinely examined in the presence of AM251, which, according to our hypothesis, primarily diminishes the inhibitory effect of CB_1_ receptors on glutamatergic neurons. The activation of presynaptic inhibitory CB_1_ receptors on GABAergic neurons that tonically inhibit glutamatergic neurotransmission should decrease the GABAergic inhibitory input leading to increases in BP and HR. Indeed, AM251 did not modify the CP55940-induced increase in BP obtained in the presence of bicuculline and enhanced that determined in the presence of L-NAME, suggesting that the CP55940 increased BP acting predominantly at inhibitory presynaptic CB_1_ receptors on GABAergic neurones or by increasing NO production. Similarly, the microinjection of the CB_1_ receptor agonist WIN55212-2 into the RVLM (Ibrahim and Abdel-Rahman 2011) or AEA into the dPAG (Dean 2011) increased BP via inhibition of brainstem GABAergic transmission and by increasing the NO level in the RVLM (Ibrahim and Abdel-Rahman 2012). Colocalization of CB_1_ receptors and NO synthase has been shown in the rat PVN (Zou et al. 2015). The glutamate-based stimulatory output of the PVN is subject to a tonic inhibition arising from GABA and NO (Pyner 2009; Kc and Dick 2010). Both bicuculline (Li et al. 2006) and AM251 (Gyombolai et al. 2012) given into the PVN caused increases in BP and/or HR, suggesting that the sympathetic tone is tonically inhibited not only by the GABAergic but also by the endocannabinergic system. The fact that the above antagonists had no influence on basal cardiovascular parameters in our study is probably related to the fact that they were administered i.v. Since L-NAME increased BP by itself, we performed additional experiments in which basal BP was increased by PGF_2α_ infusion to the level obtained after L-NAME application. Our data show that compared to the L-NAME group, AM251 further increased the CP55940-induced effect on DBP and MBP regardless of whether the basal BP was increased by L-NAME or PGF_2α_.

The AT_1_ antagonist losartan completely reversed the CP55940-induced pressor and tachycardic effects into hypotension and bradycardia. The NMDA, TP and β_2_ receptor antagonists MK801, SQ29584, and ICI118551, respectively, diminished the CP55940-induced pressor effects by about 50–60 % without affecting the CP55940-stimulated increases in HR. As shown in Fig. 5, AT_1_, TP and β_2_ receptors enhance glutamatergic transmission, which is additionally increased by the activation of NMDA receptors.

Our data suggest that AT_1_ receptors seem to play a major role in the CP55940-induced stimulatory cardiovascular responses. The particularly strong effect of losartan might result from the fact that Ang II in the PVN does not only increase the glutamatergic tone (Pyner 2009; Kc and Dick 2010; Ferguson et al. 2008; Nunn et al. 2011) but also inhibits the GABAergic tone (via an inhibitory influence on NO or GABA; Chen and Pan 2007; Nunn et al. 2011). Our results are in line with findings by Gyombolai et al. (2012) who showed that CB_1_ receptors play a role in the hypertensive effects of angiotensin II in the rat paraventricular nucleus since co-administration of AM251 together with Ang II abolished the well-known pressor effect of Ang II given into the PVN. Gyombolai et al. (2012) suggested that the Ang II-induced hypertension is connected with the activation of CB_1_ receptors, e.g. due to (1) the heterodimerization of CB_1_ and AT_1_ receptors (Rozenfeld et al. 2011) or (2) Ang II–induced endocannabinoid release that inhibits the GABAergic tone. In another study on the rat PVN (Chen and Pan 2007), a direct inhibitory effect of Ang II on GABAergic transmission, involving G_i/o_ proteins and superoxide formation, has been suggested.

Electrical stimulation of the C1 area of the rostral ventrolateral medulla increased BP in rats in a manner sensitive to intra-hypothalamic microinjection of the β_2_ antagonist ICI118551 but not to the β_1_ antagonist atenolol (Ward-Routledge et al. 1988). In addition, the PVN is richly innervated by noradrenergic nerve terminals originating from the brainstem, especially A1, A2 and A6 cell groups (Cunningham and Sawchenko 1988). It contains more β_2_- than β_1_-adrenoceptors (Rainbow et al. 1984). However, the microinjection of the β_2_-adrenoceptor agonist fenoterol did not modify BP and HR in anaesthetized rats, but authors have used one dose of the agonist (5 nmol/rat) only (Tsushima et al. 1994). On the other hand, noradrenaline microinjected into the PVN increased BP (Bachelard et al. 1992). Moreover, ICI118551 but not atenolol diminished the pressor effect of endothelin i.c.v., which may indirectly act via β_2_-adrenoceptors (Ono and Kaneko 1995).

Bilateral adrenalectomy completely reversed the pressor and tachycardic effects of CP55940 to hypotension and bradycardia of a comparable magnitude as they were before AM251 administration. Thus, we can conclude that the pressor effect of CP55940 given into the PVN is completely related to catecholamine release from the adrenal medulla but not from sympathetic nerve endings. Similarly, microinjection of a TXA_2_ mimetic into the PVN elevated plasma levels of adrenaline but had little effect on plasma levels of noradrenaline, suggesting that TP receptors are involved in the central adrenomedullary outflow in rats (Murakami et al. 2002).

Perfusion of the PVN with NMDA increased, in a manner sensitive to MK801, the local level of thromboxane B_2_ (the inactive metabolite of TXA_2_), glutamate and GABA, the plasma level of catecholamines (Okada et al. 2000; Kondo et al. 2015) and BP (Li and Pan 2007). We confirmed that microinjection of NMDA into the PVN increased BP and HR (e.g. Kawabe et al. 2008) and showed for the first time that both cardiovascular effects were diminished by AM251 given i.v. We can exclude the possibility that AM251 inhibits presynaptic inhibitory CB_1_ receptors located mainly on peripheral sympathetic nerve endings innervating resistance vessels and heart (for review, see Malinowska et al. 2010) or on central glutamatergic neurons (for review, see Schlicker and Kathmann 2001) since in both cases an increase in BP would be expected. The most plausible explanation of our data is that AM251 inhibits presynaptic inhibitory CB_1_ receptors located on central GABAergic neurons, thereby dis-inhibiting the GABAergic tone and inhibiting the NMDA-induced stimulatory effects. The fact that AM251 increases basal BP in the study by Gyombolai et al. (2012) but reduces the NMDA-induced increase in BP in the present one might be related to a normal and enhanced sympathetic tone, respectively (discussed in Grzęda et al. 2015).

## Conclusions

Stimulation of presynaptic inhibitory CB_1_ receptors on glutamatergic neurons in the PVN decreases BP and HR. Even if due to the i.v. administration of the antagonists/blockers, other locations cannot be excluded with absolute certainty that our data provide evidence that glutamatergic neurotransmission is probably increased by presynaptic facilitatory AT_1_, TP and β_2_-adrenergic heteroreceptors but inhibited by postsynaptic GABA_A_ receptors (Fig. 5). On the other hand, stimulation of presynaptic CB_1_ receptors on GABAergic neurones in the PVN might inhibit their inhibitory influence on glutamatergic neurotransmission, thereby increasing the sympathetic tone and ultimately leading to increases in BP and HR. The tone of the GABAergic neurones might in turn be increased by NO. The final effect, i.e. depression or stimulation of cardiovascular parameters, probably depends on the level of the sympathetic tone. The increase and decrease in BP (and HR) induced by the activation of CB_1_ receptors in the PVN are dependent and independent of catecholamine release from the adrenal medulla, respectively.
